# β_1_-adrenoceptor stimulation promotes LPS-induced cardiomyocyte apoptosis through activating PKA and enhancing CaMKII and IκBα phosphorylation

**DOI:** 10.1186/s13054-015-0820-1

**Published:** 2015-03-09

**Authors:** Yiyang Wang, Yuan Wang, Duomeng Yang, Xiaohui Yu, Hongmei Li, Xiuxiu Lv, Daxiang Lu, Huadong Wang

**Affiliations:** Department of Pathophysiology, School of Medicine, Jinan University, Guangzhou, 510632 Guangdong China; Key Laboratory of State Administration of Traditional Chinese Medicine of the People’s Republic of China, School of Medicine, Jinan University, Guangzhou, 510632 Guangdong China

## Abstract

**Introduction:**

Caspase activation and cardiomyocyte apoptosis have been implicated in lipopolysaccharide (LPS)-induced cardiac contractile dysfunction. We have recently demonstrated that β1-adrenoceptor (AR) activation by endogenous norepinephrine contributes to cardiomyocyte apoptosis in endotoxemic mice. Here, we further investigated the molecular mechanisms for the enhancing effect of β_1_-AR activation on LPS-induced cardiomyocyte apoptosis.

**Methods:**

The adult mouse ventricular myocytes were exposed to LPS, dobutamine, protein kinase A (PKA) inhibitor or/and nifedipine, an L-type Ca^2+^ channel blocker. Male BALB/c mice were treated with LPS or/ and β_1_-AR antagonist, atenolol. Cardiomyocyte apoptosis was determined by terminal deoxynucleotidyl transferase-mediated dUTP nick-end-labeling (TUNEL) assay and apoptosis-associated molecules were detected.

**Results:**

LPS induced apoptosis in adult mouse ventricular myocytes, dobutamine (DOB), a β_1_-AR agonist, promoted apoptosis, caspase-8, 9 and 3 activation and increased cytosolic Ca^2+^ concentration in LPS-challenged cardiomyocytes. DOB also up-regulated TNF-α expression, decreased Bcl-2 levels, promoted Bax translocation to mitochondria, mitochondrial membrane potential loss and cytochrome c release as well as IκBα, p38 MAPK, JNK and Ca^2+^/calmodulin-dependent protein kinase II (CaMKII) phosphorylation in LPS-treated cardiomyocytes. PKA inhibitor abolished the effects of DOB on caspase-9 activation, Bcl-2 levels as well as JNK and p38 MAPK phosphorylation, but not on IκBα phosphorylation, TNF-α expression and caspase-8 activation in LPS-stimulated cardiomyocytes. Pretreatment with nifedipine not only significantly blocked the enhancing effects of DOB on LPS-induced elevation in cytosolic Ca^2+^ concentration and CaMKII phosphorylation in cardiomyocytes, but also partly reversed the effects of DOB on caspase-9 and caspase-3/7 activities in LPS-treated cardiomyocytes. Furthermore, atenolol suppressed TNF-α expression, JNK, p38 MAPK and CaMKII phosphorylation, increased Bcl-2 expression, and inhibited cytochrome c release and cardiomyocyte apoptosis in the myocardium of endotoxemic mice.

**Conclusions:**

β_1_-AR activation promotes LPS-induced apoptosis through activating PKA, increasing CaMKII phosphorylation as well as enhancing IκBα phosphorylation and TNF-α expression in cardiomyocytes.

## Introduction

Myocardial dysfunction is a common complication that greatly contributes to the mortality of sepsis in both patients and experimental models of lipopolysaccharide (LPS)-induced endotoxemia [1,2]. Accumulating evidence has indicated that cardiomyocyte caspase activation plays an important role in myocardial dysfunction in patients and various animal models of sepsis, such as endotoxemia [3-6]. LPS can activate caspases in cardiomyocytes *in vivo* and *in vitro* [5,7,8], and caspase-3 activation not only induces end-stage nuclear apoptosis, cleavage of cardiac myofilament proteins and sarcomere disorganization [5], but also blunts contractile response of ventricular myocytes to norepinephrine [9]. On the contrary, caspase-3 inhibition ameliorates LPS-induced myocardial contractile dysfunction [10,11]. Clearly, it is very important to identify and block the initiating apoptotic stimuli or inhibit apoptotic pathways in cardiomyocytes for treating sepsis-induced myocardial dysfunction.

To date LPS-induced cardiomyocyte apoptosis has been attributed to increased production of TNF-α, macrophage migration inhibitory factor and reactive oxygen species [7,12-15]. Although it is well-documented that plasma norepinephrine levels are significantly elevated in the setting of sepsis [16] and norepinephrine alone can directly stimulate cardiomyocyte apoptosis by activating the β-adrenoceptor (AR) [17,18], little is known about the causative contribution of endogenous norepinephrine in sepsis-induced cardiomyocyte apoptosis. Recently, our preliminary results demonstrated that both exhaustion of cardiac endogenous norepinephrine and blockade of β_1_-AR significantly suppressed myocardial caspase-3 activation in LPS-challenged mice [19], suggesting that β_1_-AR activation by endogenous norepinephrine may be a significant contributor to cardiomyocyte apoptosis during endotoxemia. However, the molecular mechanisms for this enhancing effect of β_1_-AR activation on cardiomyocyte apoptosis during endotoxemia have not been identified. It is well-known that β_1_-AR activation induces pro-apoptotic signals through protein kinase A (PKA) and Ca^2+^/calmodulin-dependent protein kinase II (CaMKII) activation in cardiomyocytes [20,21]. Therefore, we hypothesize that PKA and CaMKII activation mediate the enhancing effect of β_1_-AR activation on LPS-induced cardiomyocyte apoptosis. Here, we used adult mouse ventricular myocytes and a mouse model of endotoxemia to test this hypothesis. The data demonstrate that cardiomyocyte β_1_-AR activation is a significant contributor to cardiomyocyte apoptosis during endotoxemia. Stimulation of β_1_-AR activates PKA, increases CaMKII and IκBα phosphorylation as well as TNF-α expression, in turn enhances caspase-9 and 8 activities, and promotes LPS-induced cardiomyocyte apoptosis.

## Materials and methods

### Animals

Male BALB/c mice were obtained from the medical laboratory animal center of Guangdong province and acclimatized to laboratory conditions for at least 6 days following their arrival. All experiments with animals were conducted in compliance with the guide for the Care and Use of Laboratory Animals published by the US National Institutes of Health, and approved by the Animal Care and Use Committee at Jinan University School of Medicine. Under necessary conditions, the mice were anesthetized with pentobarbital (100 mg/kg), the adequacy of the anesthesia was monitored, and every effort was made to minimize suffering.

### Animal procedures

Male BALB/c mice were randomized into different groups and received intraperitoneal injection with vehicle or atenolol (Sigma-Aldrich, St Louis, MO, USA) at a dose of 10 mg/kg. LPS, 20 mg/kg; *Escherichia coli*, 055:B5 (Sigma-Aldrich) or normal saline was administered intraperitoneally 1 hour after treatment with atenolol. At 2, 6 or 12 hours after LPS injection, the serum and hearts of mice were collected for further analysis.

### Adult mouse ventricular myocyte isolation and culture

Adult mouse ventricular myocytes were obtained from hearts of male BALB/c mice using Langendorff perfusion apparatus as described previously [22]. Cardiomyocytes (30 to 50 cells/mm^2^) were plated on fibronectin-coated polystyrene tissue culture dishes. Plated cells were incubated in serum-free culture medium (DMEM) supplemented with HEPES (25 mM), bovine serum albumin (BSA, 0.2%), creatine (5 mM), L-carnitine (2 mM), taurine (5 mM) and 0.1% penicillin-streptomycin at 37°C under a 5% CO_2_-95% air atmosphere. Two hours after plating, the culture medium was changed to remove unattached dead cells and the viable cardiomyocytes were incubated overnight under the same culture conditions.

### Cardiomyocyte treatment

Adult mouse ventricular myocytes, cultured for 24 hours, were treated with β_1_-AR agonist, dobutamine (DOB, 0.02, 0.2, 2 and 20 μM; Sigma-Aldrich) and/or LPS (1, 10, 100 and 1,000 ng/mL; Sigma-Aldrich). To inhibit PKA or L-type Ca^2+^ channel, KT5720 (5 μM; Sigma-Aldrich) or nifedipine (NIF, 1 μM; Sigma-Aldrich) was added for 1 hour prior to DOB and/or LPS treatment.

### Determination of caspase-3/7 activity

The caspase-3/7 activity in cardiomyocytes or myocardium was detected by using the Apo-ONE® homogeneous caspase-3/7 assay kit (Promega Corp., Madison, WI., USA) according to the manufacturer’s instructions.

### Determination of mitochondrial membrane potential

Mitochondrial membrane potential was visualized in mouse ventricular myocytes stained with 5, 5', 6, 6'-tetrachloro-1, 1', 3, 3'-tetraethylbenzimidazolcarbocyanine iodide (JC-1) by using a laser scanning confocal microscope, as described previously [23]. JC-1 can be accumulated and aggregates (red fluorescence) in normal mitochondria. The loss of mitochondrial membrane potential prevents JC-1 entry into mitochondria, monomeric JC-1 (green fluorescence) remains in the cytosol. The mouse ventricular myocytes from different treatment groups were washed and incubated with JC-1 at 37°C for 15 minutes, then washed and mounted on the confocal microscopy for imaging. The ratio of monomeric to aggregated JC-1 fluorescence intensity was used to quantify changes in mitochondrial membrane potential, and the increased ratio represents the decrease in mitochondrial membrane potential.

### Terminal deoxynucleotidyl transferase-mediated dUTP nick-end-labeling (TUNEL) assay

Cardiomyocyte apoptosis was determined by TUNEL assay using an *in situ* cell death detection kit (Roche Applied Science, Indianapolis, IN, USA). Briefly, cardiomyocytes were fixed with 4% paraformaldehyde in PBS for 40 minutes at room temperature, washed and permeabilized with 0.1% Triton X-100 for another 5 minutes at 4°C. The fixed cells were incubated with 1:50 dilution of anti-cardiac troponin I (cTnI; Abcam, Cambridge, UK) at 37°C for 1 hour, and subjected to the TUNEL assay according to the manufacturer’s instructions. Then, the cells were washed three times with PBS, incubated with 1:1,000 dilution of secondary antibody conjugated with Alexa Fluor® dyes (Invitrogen, Life Technologies, Grand Island, NY, USA) for 1 hour in the dark. Finally, the cells were incubated with 1:400 dilution of 4',6-diamidino-2-phenylindole (DAPI) for 15 minutes, washed and mounted on coverslips with antifade mounting medium. For *in vivo* study, tissue sections (5 μm) from frozen cardiac tissues were fixed with 4% paraformaldehyde for 20 minutes and permeabilized in 0.1% Triton X-100 in 0.1% sodium citrate for 2 minutes at 4°C. Triple staining with anti-cardiac troponin I, TUNEL and DAPI was also performed. Fluorescent images were taken in 10 random high-powered fields using laser confocal microscopy. The numbers of TUNEL-positive cardiomyocytes and of total cardiomyocyte nuclei were counted, and the apoptotic index (AI) of cardiomyocytes was expressed as a percentage of TUNEL-positive cells to total cells.

### Caspase-8 and caspase-9 activity assay

After indicated treatments, the cardiomyocytes were subjected to caspase-8 and caspase-9 activity measurement using Caspase-Glo assay kit (Promega). Briefly, the plates containing cells were removed from the incubator. Caspase-Glo reagent (100 μl) was added to each well and gently mixed. The plate was incubated for 2 hours at room temperature. Then, the luminescence of each sample was measured in a plate-reading luminometer.

### Measurement of TNF-α and nuclear NF-κB activity

Cardiac and serum TNF-α was detected using a mouse TNF-α enzyme-linked immunosorbent assay (ELISA) kit (R&D Systems, Inc, Minneapolis, MN, USA) according to the manufacturer’s instructions. Cardiomyocyte nuclear NF-κB activity was measured using a NF-kB (p65) Transcription Factor Assay kit (Cayman Chemical, Ann Arbor, MI, USA) as previously described [24].

### Determination of cytosolic Ca^2+^ concentration

Adult mouse ventricular myocytes were loaded with Ca^2+^ sensitive indicator fluo-4 AM (2 μM; Invitrogen) in the dark at 37°C for 60 minutes. After the loading, cells were washed three times with dye-free buffer solution for 30 minutes. Fluo-4-loaded cells were excited with an argon laser beam (at a wavelength of 488 nm), and the emitted fluorescence at 520 nm from defined regions within the cells was monitored over time on a confocal laser scanning microscope. The baseline fluo-4 fluorescence intensity at the beginning of each experiment and fluo-4 fluorescence intensity 3 hours after treatment with DOB (0.02 μM), or/and LPS (10 ng/mL) were measured, changes in cytosolic Ca^2+^ concentration were expressed as a percent relative to the baseline fluorescence intensity, and the data were normalized to controls.

### Western blotting analysis

Cytosolic and mitochondrial proteins were harvested from cardiomyocytes or heart homogenates, and protein concentration detected. The levels of specific proteins were determined by western blotting assay, as previously described [23]. The following primary antibodies were used: anti-glyceraldehyde-3-phosphate dehydrogenase (GAPDH), anti-voltage-dependent anion channel (VDAC), anti-c-jun NH2-terminal kinase (JNK), anti-extracellular signal-regulated kinase (ERK), anti-p38 MAPK (mitogen-activated protein kinase), anti-IκBα, anti-Ca2+/calmodulin-dependent protein kinase II (CaMKII), anti-p-JNK, anti-p-ERK, anti-p-p38 MAPK, anti-p-IκBα, anti-p-CaMKII, anti-Bcl-2, anti-cytochrome c (Cyt c), anti-Bax and anti-TNF-α antibody (Cell Signaling technology, Beverly, MA, USA). GAPDH or VDAC was used to normalize protein loading.

### Statistical analysis

All data were presented as mean ± standard error of the mean (SEM). One-way analysis of variance (ANOVA) followed by Bonferroni post hoc analysis was performed for comparison among multiple groups. Values of *P* <0.05 were considered to be statistically significant.

## Results

### Dobutamine promotes LPS-induced caspase 3/7 activation and nuclear apoptosis in adult mouse cardiomyocytes

**Figure 1:**
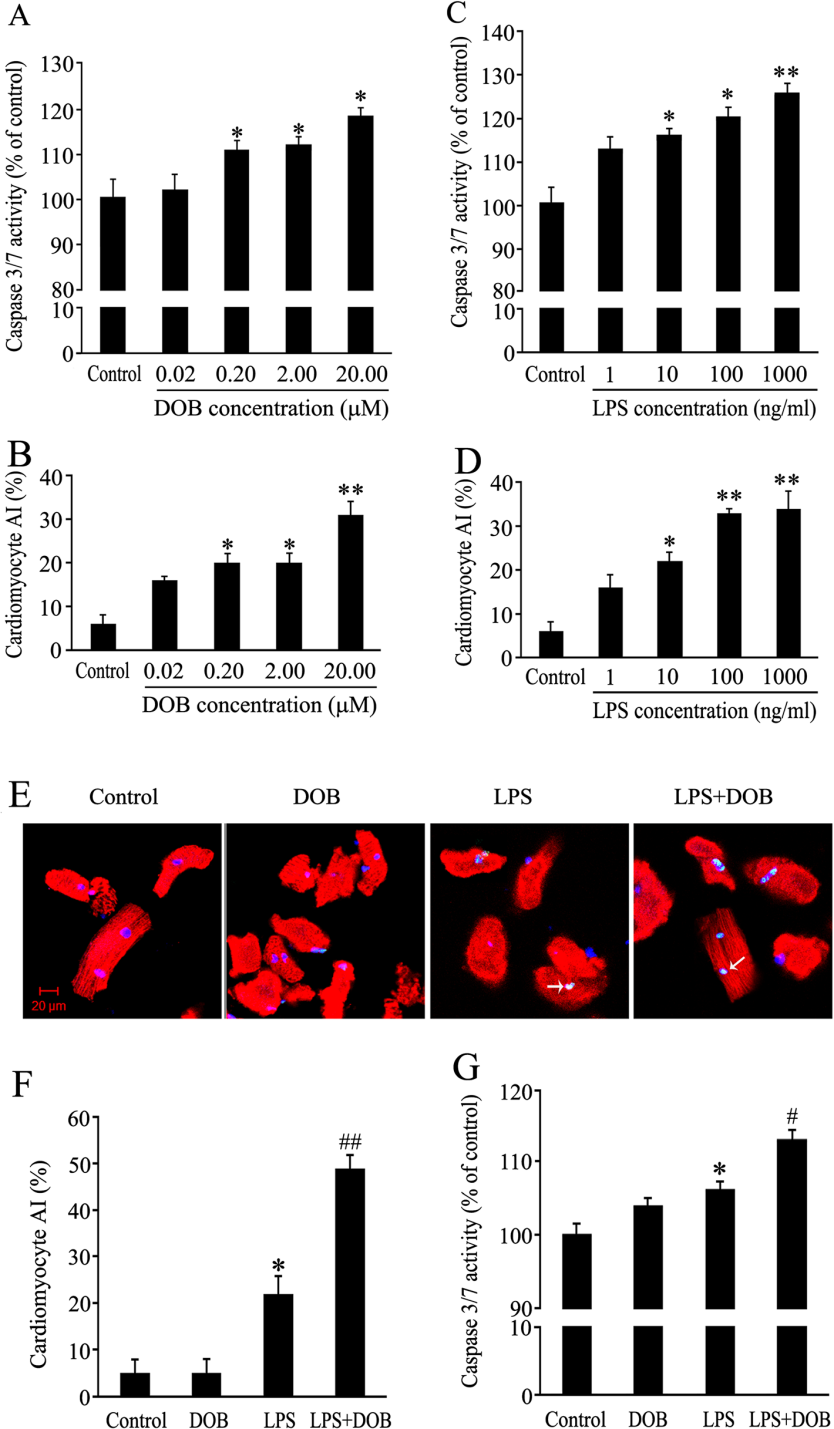
**Caspase 3/7 activity and apoptotic index of cardiomyocytes treated with dobutamine (DOB) and lipopolysaccharide (LPS). (A,B)** Adult mouse ventricular myocytes treated with DOB at various doses for 24 hours. **(C,D)** Adult mouse ventricular myocytes exposed to LPS at various concentrations for 24 hours. **(E)** Representative confocal images of TUNEL assay (green) of adult mouse ventricular myocytes treated with 0.02 μM DOB or/and 10 ng/ml LPS for 24 hours; all cardiomyocytes were stained with anti-cardiac troponin I (cTnI) antibody (red) and total nuclei with 4', 6-diamidino-2-phenylindole (DAPI, blue). White arrowheads indicate apoptotic cardiomyocytes (scale bar = 20 μm). **(F,G)** AI and caspase-3/7 activity in cardiomyocytes treated with 0.02 μM DOB or/and 10 ng/mL LPS for 24 hours. Mean ± standard error of the mean (n = 4 to 6). ^*^ *P* <0.05, **P <0.01 compared with the control group; ^#^ *P* <0.05, ^##^ *P* <0.01 compared with the LPS group.

To assess the direct effect of cardiomyocyte β_1_-AR activation on LPS-stimulated cardiomyocyte apoptosis, we performed TUNEL assay and examined the caspase 3/7 activity in isolated adult mouse ventricular myocytes treated with DOB, a β_1_-AR agonist, or/and LPS at various concentrations for 24 hours. DOB (0.02 to 20.00 μM) and LPS (1, 10, 100 and 1,000 ng/mL) induced caspase 3/7 activation and nuclear apoptosis in cardiomyocytes in a dose-dependent manner (Figure 1A, B, C and D). Compared with control cardiomyocytes, caspase 3/7 activity and the percentage of apoptotic cells were increased in LPS (10 ng/mL)-treated cardiomyocytes. Although treatment with DOB at a dose of 0.02 μM had no significant effect on either cardiomyocyte caspase 3/7 activity or nuclear apoptosis, it markedly promoted caspase 3/7 activation and nuclear apoptosis in cardiomyocytes exposed to 10 ng/mL LPS (Figure 1E, F and G).

### Dobutamine enhances activation of caspase-8 and caspase-9 in LPS-treated adult mouse cardiomyocytes

**Figure 2:**
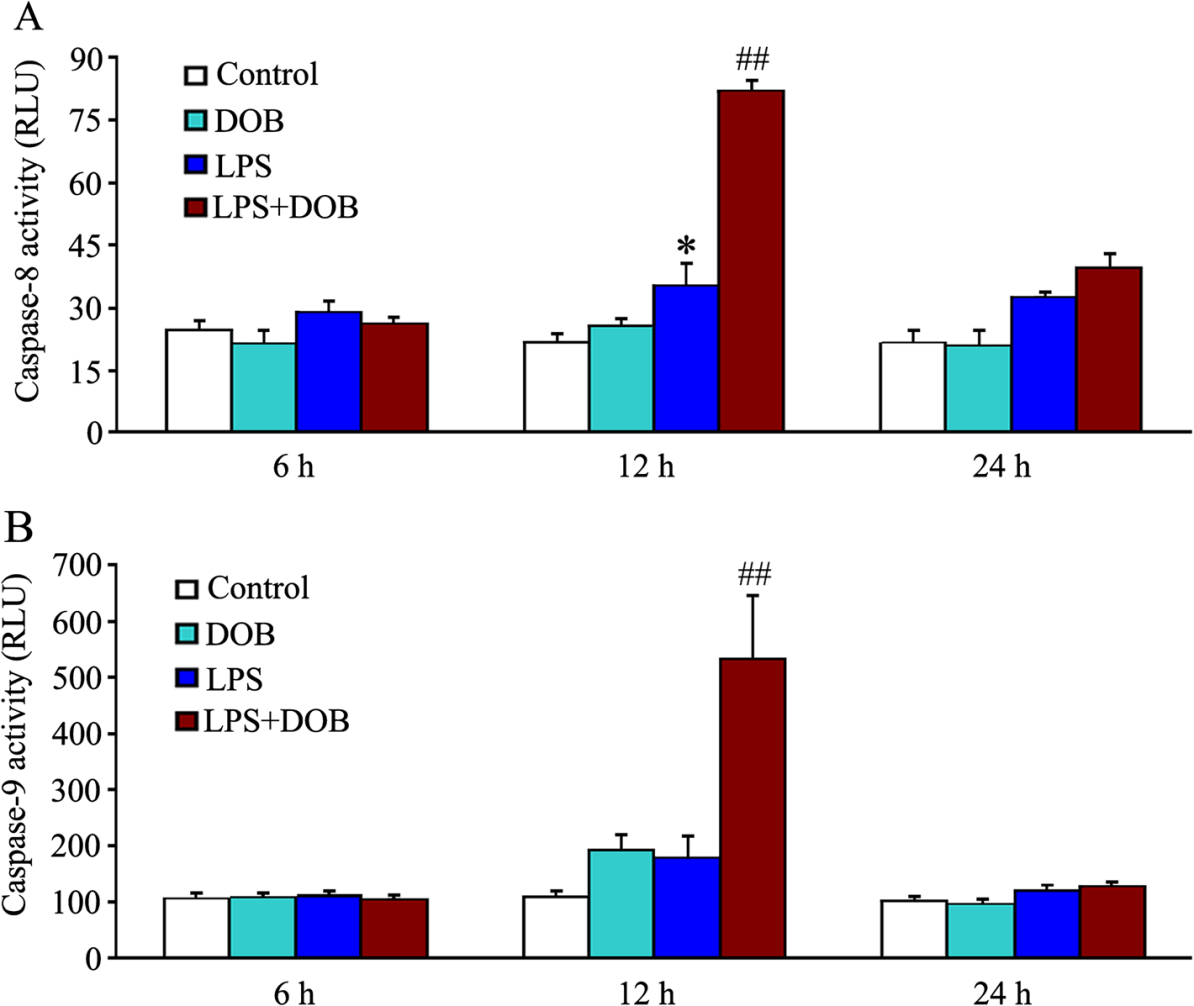
**Dobutamine (DOB) enhances caspase-8 and caspase-9 activation in lipopolysaccharide (LPS)-treated adult mouse cardiomyocytes.** Caspase-8 **(A)** and caspase-9 **(B)** activities were detected in cardiomyocytes treated with 0.02 μM DOB or/and 10 ng/mL LPS for 6, 12 or 24 hours. Mean ± standard error of the mean (n = 4). ^*^ *P* <0.05 compared with the control group; ^##^ *P* <0.01 compared with the LPS group.

Caspase-8 activity in cardiomyocytes treated with 10 ng/mL LPS alone significantly increased at 12 hours after LPS exposure compared with control cells (Figure 2A). Stimulation with 0.02 μM DOB for 6, 12 or 24 hours did not induce caspase-8 activation. However, caspase-8 activity was markedly elevated when 10 ng/mL LPS was administered with 0.02 μM DOB compared to the LPS-only group. In addition, the slight increase in cardiomyocyte caspase-9 activity 12 hours after LPS stimulation was not statistically significant. In contrast, caspase-9 activity was significantly higher in cardiomyocytes at 12 hours after stimulation in the DOB plus LPS-treated group than in the LPS-only group (Figure 2B).

### Dobutamine promotes LPS-induced TNF-α release and NF-κB activation, as well as IκBα, JNK and p38 MAPK phosphorylation in adult mouse cardiomyocytes

**Figure 3:**
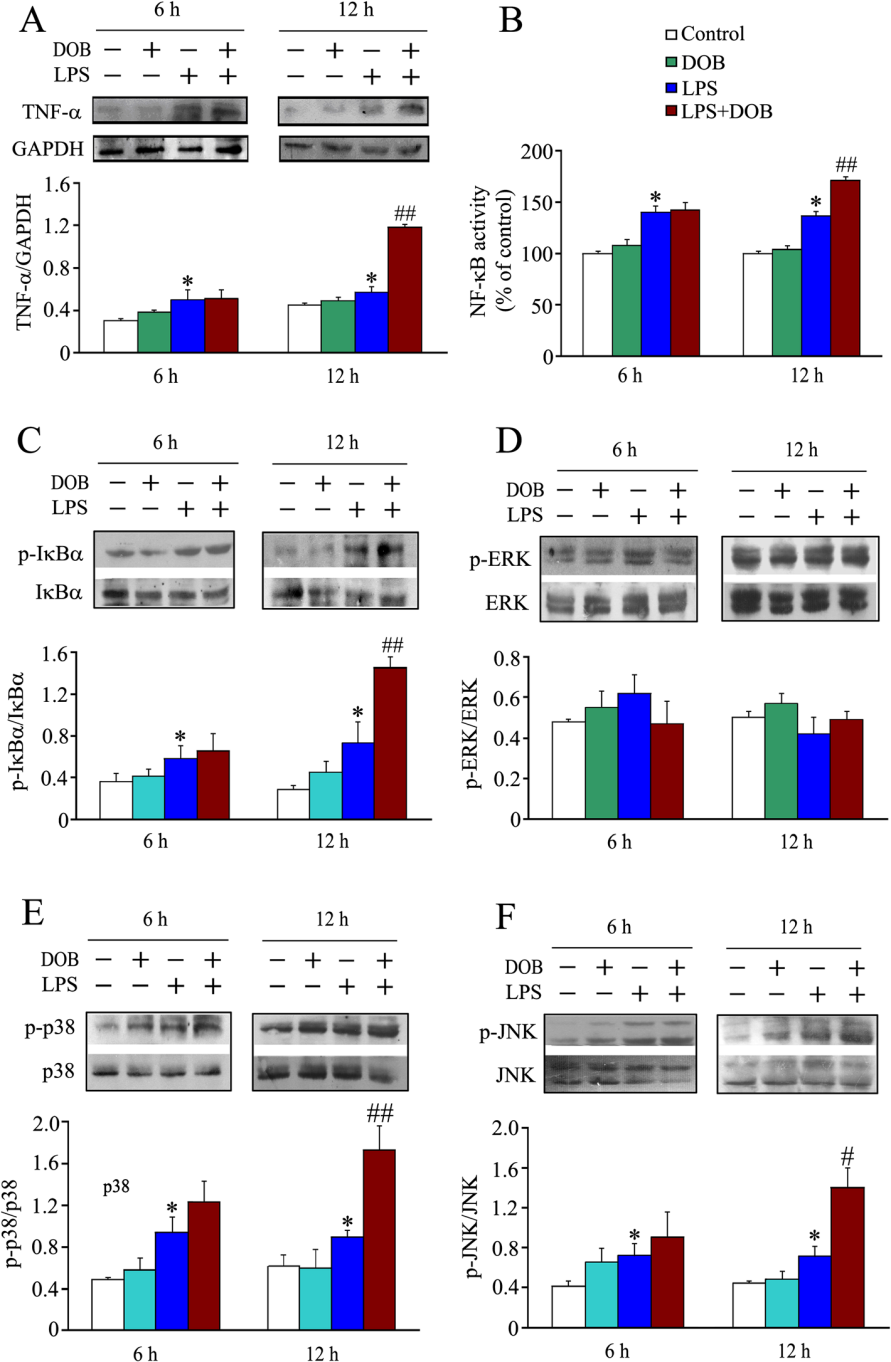
**Dobutamine promotes TNF-α expression and phosphorylation of IκBα, p38 mitogen-activated protein kinase (MAPK) and c-jun NH2-terminal kinase (JNK) in lipopolysaccharide (LPS)-treated cardiomyocytes.** Adult mouse ventricular myocytes were treated with dobutamine (DOB, 0.02 μM), LPS (10 ng/mL), their combination, or vehicle for 6 hours and 12 hours. The total cardiomyocyte lysates and nuclear proteins were prepared. **(A)** The expression of TNF-α in cardiomyocytes was detected by western blot (mean ± standard error of the mean (SEM); n = 6). **(B)** NF-kB p65 DNA binding activity in nuclear proteins was examined using Cayman’s NF-kB (p65) Transcription Factor Assay kit, and is expressed as percent changes relative to control (mean ± SEM; n = 6). The levels of p-IκBα/IκBα **(C)**, p-ERK/ERK **(D)**, p-p38 MAPK/ p38 MAPK **(E)** and p-JNK/JNK **(F)** were examined by western blotting of whole cell lysates with specific antibodies, respectively (mean ± SEM; n = 3). ^*^ *P* <0.05 compared with the control group; ^#^ *P* <0.05; ^##^ *P* <0.01 compared with the LPS group.

It has been reported that TNF-α play a critical role in LPS-induced cardiomyocyte apoptosis [7,12]. Therefore, we determined cardiomyocyte TNF-α production at 6 hours and 12 hours after DOB or/and LPS treatment. As shown in Figure 3A, at 12 hours after LPS challenge, expression of TNF-α in cardiomyocytes was elevated in the 10 ng/mL LPS group compared to controls, and 0.02 μM DOB further increased LPS-induced TNF-α expression in cardiomyocytes. It is well-known that LPS stimulates TNF-α expression through MyD88-dependent NF-κB and MAPK activation. We further observed the effects of DOB or/and LPS on NF-κB activation, inhibitor of κBα (IκBα), JNK, ERK and p38 MAPK phosphorylation in adult mouse cardiomyocytes. LPS increased nuclear NF-κB activity, IκBα, JNK and p38 MAPK phosphorylation, but not ERK phosphorylation in cardiomyocytes at 12 hours after LPS exposure. Treatment with DOB significantly promoted LPS-induced NF-κB activation as well as IκBα, JNK and p38 MAPK phosphorylation in cardiomyocyte at 12 hours after LPS treatment (Figure 3B-F).

### Dobutamine decreases Bcl-2 levels, increases mitochondrial Bax contents, reduces mitochondria membrane potential and promotes cytochrome c release in LPS-treated adult mouse cardiomyocytes

**Figure 4:**
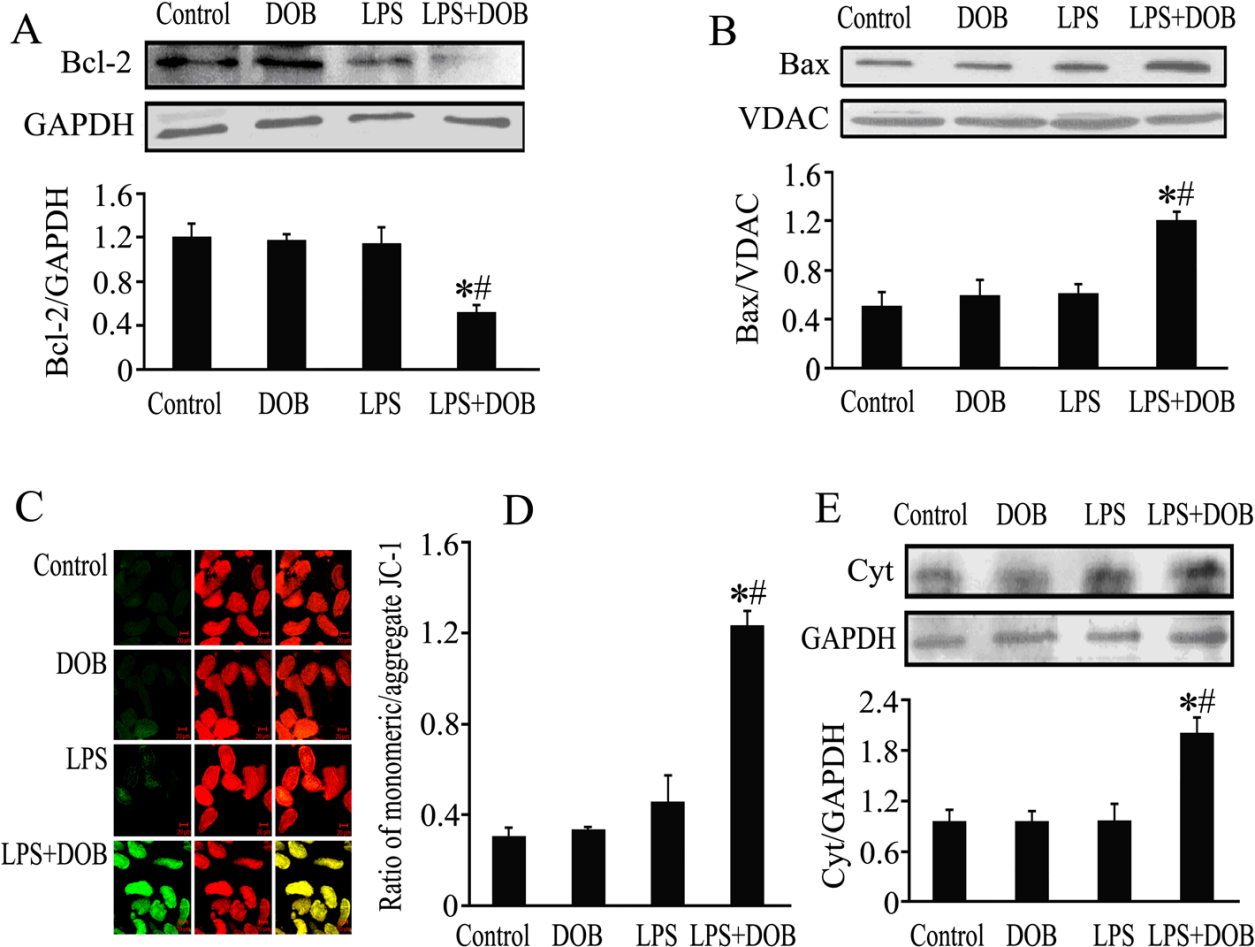
**Effects of dobutamine (DOB) and lipopolysaccharide (LPS) on cardiomyocyte Bcl-2 levels, mitochondrial Bax contents, mitochondrial membrane potential and cytochrome c release.** Adult mouse ventricular myocytes were treated with DOB (0.02 μM), LPS (10 ng/mL), their combination, or vehicle for 12 hours. **(A)** Bcl-2 levels were analyzed by western blotting of whole cell lysates. **(B)** Mitochondrial Bax protein contents were detected by western blotting of mitochondrial proteins and voltage-dependent anion channel (VDAC) was immunoblotted as a mitochondrial marker. **(C)** Confocal images of JC-1 fluorescence. Mitochondrial membrane potential was visualized in ventricular myocytes stained with JC-1, an indicator of mitochondrial function, green fluorescence indicates monomeric JC-1 (left), red fluorescence represents aggregate JC-1 (middle) and merged channels are shown (right): scale bar = 20 μm. **(D)** The ratio of monomeric and aggregated JC-1, indicating changes in mitochondrial membrane potential. **(E)** Cytosolic cytochrome c (Cyt c) contents were analyzed by western blotting of cytosolic proteins and GAPDH was used as an internal control: mean ± standard error of the mean (n = 3). ^*^ *P* <0.05 compared with the control group; ^#^ *P* <0.05 compared with the LPS group.

It is well-known that the mitochondria-dependent apoptotic pathway also mediates cardiomyocyte apoptosis. We further observed the effects of DOB and LPS on this apoptotic pathway. As shown in Figure 4, treatment with 10 ng/mL LPS for 12 hours did not induce evident changes in Bcl-2, cytosolic Cyt c and mitochondrial Bax protein levels and mitochondrial membrane potential compared with controls. In contrast, at 12 hours after co-stimulation with DOB (0.02 μM), cytosolic Cyt c and mitochondrial Bax levels were markedly higher in the DOB plus LPS group than those in the LPS group, and Bcl-2 levels and mitochondrial membrane potential were significantly lower in the DOB plus LPS group than those in the LPS group.

### PKA inhibitor abolishes the effect of DOB on caspase-9 activation and Bcl-2 level as well as JNK and p38 MAPK phosphorylation, but not on IκBα phosphorylation and caspase-8 activation in LPS-stimulated cardiomyocytes

**Figure 5:**
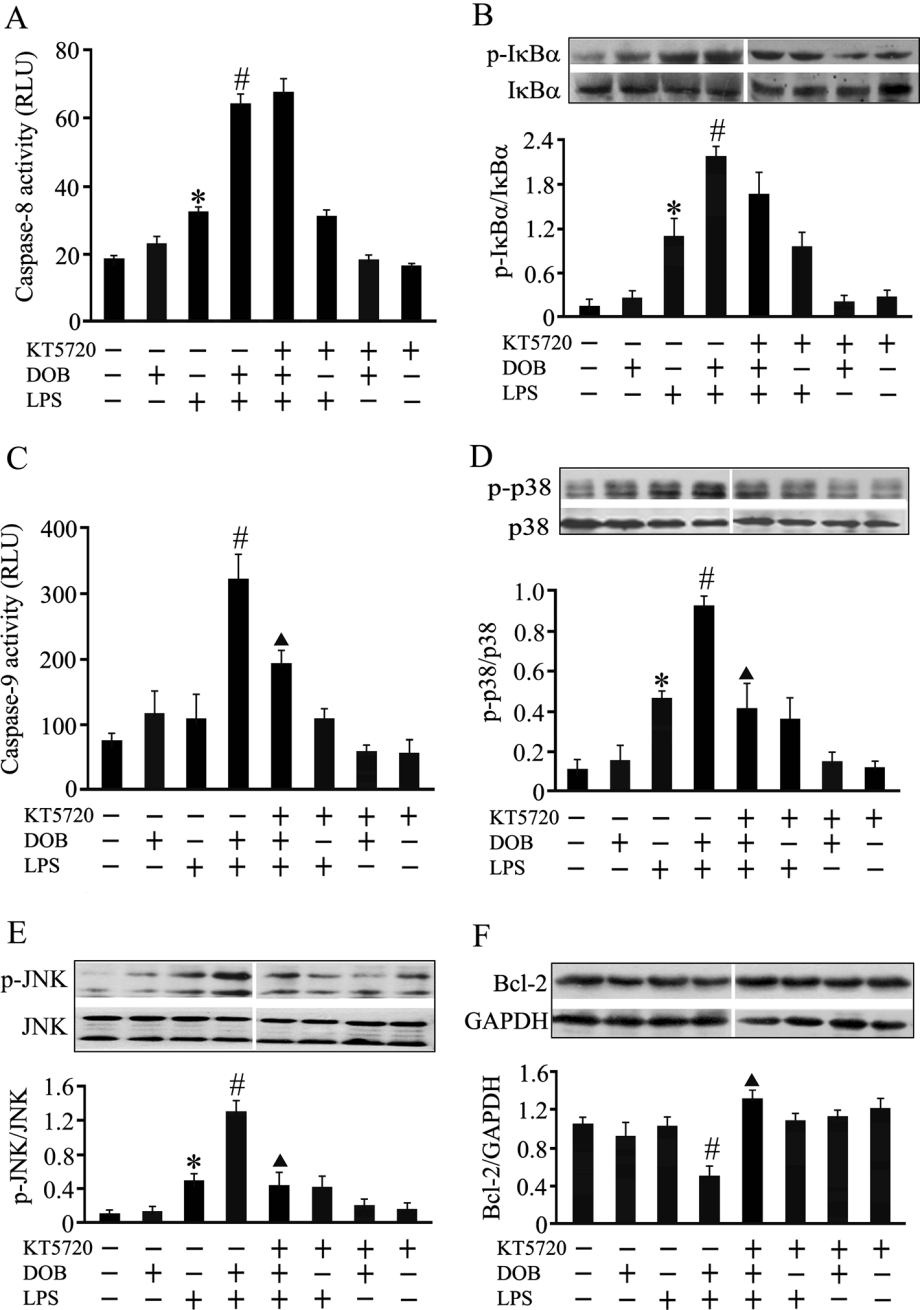
**Effects of KT5720, a protein kinase A (PKA) inhibitor, on apoptosis-associated molecules in dobutamine (DOB) and lipolysaccharide (LPS)-treated cardiomyocytes.** Adult mouse ventricular myocytes were pretreated with KT5720 (5 μM) or vehicle for 1 hour, and then exposed to DOB (0.02 μM), LPS (10 ng/mL), their combination, or vehicle for 12 hours. Caspase-8 **(A)** and caspase-9 **(C)** activities were detected 12 hours after DOB and LPS treatment (mean ± standard error of the mean (SEM); n = 8). IκBα **(B),** p38 mitogen-activated protein kinase (MAPK) **(D)** and c-jun NH2-terminal kinase (JNK) **(E)** phosphorylation, as well as Bcl-2 **(F)** protein expression in cardiomyocytes, were also examined 12 hours after DOB and LPS treatment using western blotting (mean ± SEM; n = 3). The results showed that KT5720 reversed the effects of dobutamine (DOB) on caspase-9 activation, Bcl-2 expression as well as p38 MAPK and JNK phosphorylation, but not on caspase-8 activation and IκBα phosphorylation in LPS-challenged cardiomyocytes. ^*^ *P* <0.05 compared with the control group; ^#^ *P* <0.05 compared with the LPS group; ^▲^ *P* <0.05 compared with the DOB plus LPS group.

It has been demonstrated that β_1_-AR activation induces pro-apoptotic signals through PKA activation [20]. To further investigate the role of PKA in the mechanisms for enhancing the effect of DOB on LPS-induced cardiomyocyte apoptosis, we used a PKA inhibitor, KT5720, to treat adult mouse cardiomyocytes 1 hour prior to DOB or/and LPS stimulation, and examined apoptosis-associated molecules in cardiomyocytes. As depicted in Figure 5, caspase-8 activity showed a marked increase 12 hours after stimulation with LPS, but the slight increase in cardiomyocyte caspase-9 activity 12 hours after LPS stimulation was not statistically significant compared with control. These effects of LPS on cardiomyocytes were enhanced by DOB treatment. Furthermore, inhibition of PKA activation with KT5720 reversed the enhancing effects of DOB on caspase-9, but not caspases-8 activation in LPS-challenged cardiomyocytes, while pretreatment with KT5720 alone did not affect caspase-8 and caspase-9 activation in control and LPS-stimulated cardiomyocytes (Figure 5A and C). Similarly, DOB also promoted LPS-induced IκBα, JNK and p38MAPK phosphorylation 12 hours after LPS exposure in cardiomyocytes. Except for IκBα phosphorylation, these effects of DOB in LPS-treated cardiomyocytes were completely blocked by KT5720 pretreatment (Figure 5B, D, E). In addition, DOB significantly reduced Bcl-2 protein level in LPS-treated cardiomyocytes, which was prevented by KT5720 treatment (Figure 5F).

### Dobutamine enhances LPS-induced elevation in cytosolic Ca^2+^ concentration and CaMKII phosphorylation, which were completely blocked by pretreatment with nifedipine

**Figure 6:**
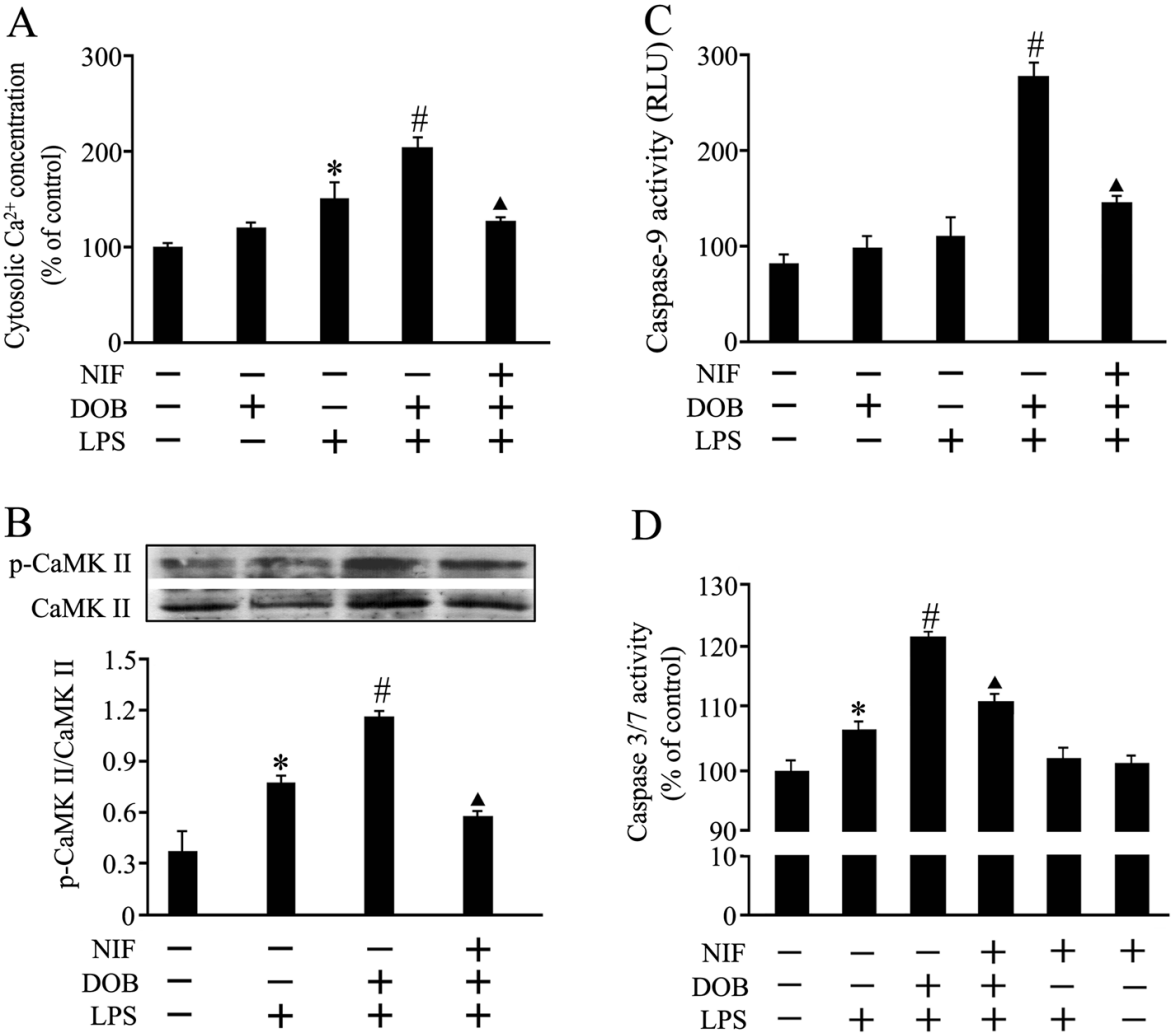
**Effects of dobutamine (DOB), lipolysachharide (LPS) and nifedipine (NIF) on cytosolic Ca** ^**2+**^ **, calmodulin-dependent protein kinase II (CaMKII) and caspase activation in cardiomyocytes.** Adult mouse ventricular myocytes were pretreated with NIF (1 μM)**,** a calcium channel blocker, or vehicle for 1 hour, and then exposed to DOB (0.02 μM), LPS (10 ng/mL), their combination or vehicle for 3, 12 or 24 hours. **(A,B)** DOB enhanced LPS-induced cytosolic Ca^2+^ concentration elevation (mean ± standard error of the mean (SEM); n = 8) and calmodulin-dependent protein kinase II (CaMKII) phosphorylation (mean ± SEM; n = 4) 3 hours and 12 hours after LPS treatment, respectively, both of which were blocked by NIF pretreatment. **(C,D)** NIF partly abolished the enhancement effects of DOB on LPS-stimulated caspase-9 (12 hours after DOB and LPS treatment) and caspase 3/7 (24 hours after DOB and LPS treatment) activation in cardiomyocytes (mean ± SEM; *n* = 8). ^*^ *P* <0.05 compared with the control group; ^#^ *P* <0.05 compared with the LPS group; ^▲^ *P* <0.05 compared with the DOB plus LPS group.

It has been shown that PKA-independent increases in cytosolic Ca^2+^ concentration and CaMKII activity contribute to cardiomyocyte apoptosis induced by sustained β_1_-AR stimulation [21]. In order to determine the roles of the Ca^2+^ and CaMKII signaling pathway in the promoting effect of β_1_-AR stimulation on LPS-provoked cardiomyocyte apoptosis, we used NIF (1 μM), an L-type Ca^2+^ channel antagonist, to treat cardiomyocytes 1 hour prior to DOB and/or LPS treatment, and examined cytosolic Ca^2+^ concentration and CaMKII phosphorylation. As shown in Figure 6A and B, LPS treatment not only significantly increased cytosolic Ca^2+^ concentration, but also elevated CaMKII phosphorylation in cardiomyocytes, both of which were enhanced by co-stimulation with DOB; these effects of DOB in LPS-treated cardiomyocytes were completely blocked by pretreatment with NIF.

### Nifedipine partly abolishes the enhancing effects of dobutamine on caspase-9 and caspase-3/7 activities in LPS-treated cardiomyocytes

As shown in Figure 6C and D, DOB significantly increased caspase-9 and caspase-3/7 activities in LPS-stimulated cardiomyocytes, these effects of DOB were partly reversed with NIF pretreatment. NIF exposure alone failed to affect caspase-9 (data not shown) and caspase-3/7 activities in control and LPS-treated cardiomyocytes.

### Blockade of β_1_-AR decreases myocardial TNF-α expression, JNK, p38 MAPK and CaMKII phosphorylation, restores Bcl-2 level and reduces cytochrome c release and cardiomyocyte apoptosis in LPS-challenged mice

**Figure 7:**
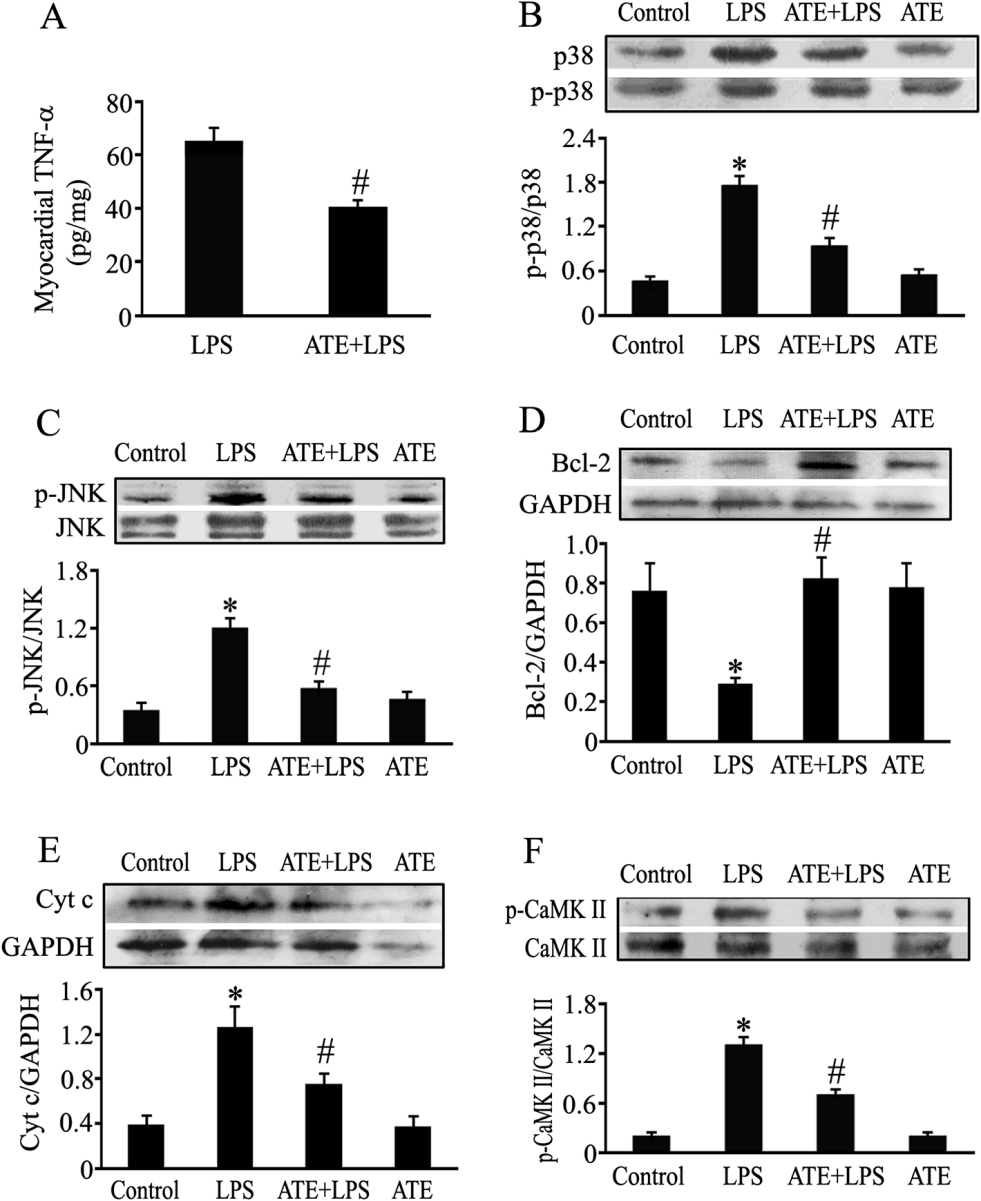
**Effect of atenolol pretreatment on myocardial apoptosis-associated molecules in endotoxemic mice.** Animals received intraperitoneal injection of normal saline or atenolol (ATE, 10 mg/kg) 1 hour before normal saline or 20 mg/kg LPS administration. **(A)** Myocardial TNF-α content was determined by ELISA at 2 hours after LPS challenge (mean ± standard error of the mean (SEM); n = 10). **(B-F)** Myocardial p38 mitogen-activated protein kinase (MAPK) and c-jun NH2-terminal kinase (JNK) phosphorylation, Bcl-2 contents, Cyt c release and calmodulin-dependent protein kinase II (CaMKII) phosphorylation were determined using western blotting at 2 hours after lipolysaccharide (LPS) challenge (mean ± SEM; n = 5 to 6). ^*^ *P* <0.05 compared with the control group; ^#^ *P* <0.05 compared with the LPS group.

**Figure 8:**
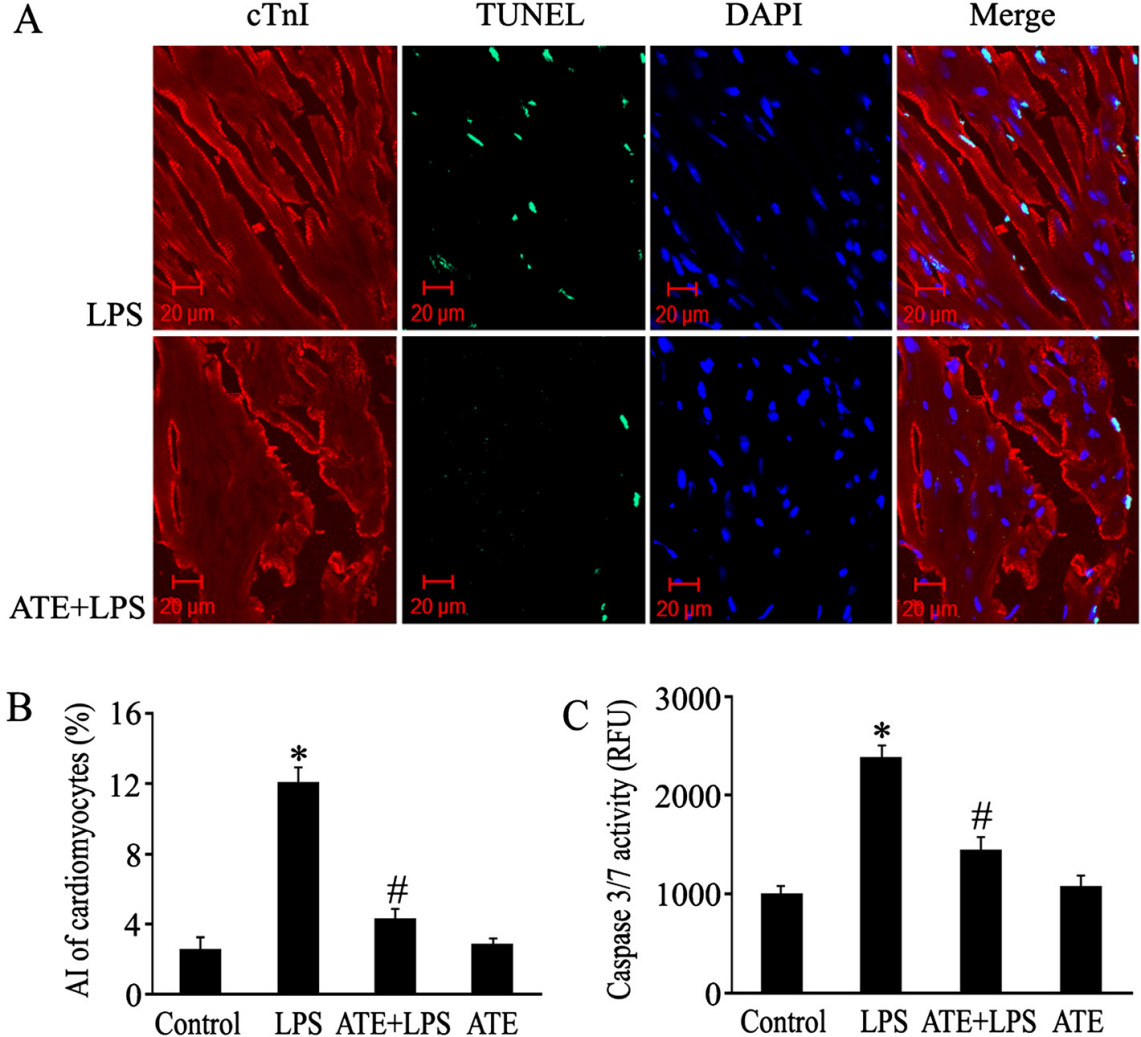
**Atenolol (ATE) inhibits cardiomyocyte apoptosis in lipolysaccharide (LPS)-challenged mice.** Animals received intraperitoneal injection of normal saline or ATE (10 mg/kg) 1 hour before normal saline or 20 mg/kg LPS administration. At 12 hours after LPS administration, cardiomyocyte apoptosis was examined by TUNEL assay. **(A)** Representative photomicrographs of TUNEL assay (green) in the hearts from LPS and ATE plus LPS groups. Total nuclei were stained with DAPI (blue) and cardiomyocytes with anti-cTnI antibody (red). **(B)** Apoptotic index (AI) of cardiomyocytes (mean ± standard error of the mean (SEM); n = 8). **(C)** Cardiac caspase 3/7 activity at 6 hours after LPS administration (mean ± SEM; n = 8). ^*^ *P* <0.05 compared with the control group; ^#^ *P* <0.05 compared with the LPS group.

In order to confirm the molecular mechanisms that β_1_-AR activation promoted LPS-induced cardiomyocyte apoptosis during endotoxemia, we further investigated the effects of ATE, β_1_-AR antagonist, on the above apoptosis-associated molecules in the myocardium of LPS-challenged mice. As shown in Figure 7, compared with control mice, myocardial TNF-α levels in LPS-treated mice were significantly increased at 2 hours after LPS injection, and Cyt c release as well as JNK, p38 MAPK and CaMKII phosphorylation were also elevated in LPS-challenged mice. On the contrary, myocardial Bcl-2 protein levels were reduced in LPS-challenged mice at 2 hours after LPS injection. These effects of LPS were inhibited by ATE pretreatment. In addition, treatment with LPS for 12 hours significantly increased cardiomyocyte apoptosis as assessed by TUNEL staining. In contrast, pretreatment with ATE at 1 hour before LPS challenge markedly suppressed LPS-induced cardiomyocyte apoptosis (Figure 8A and B). Similarly, the myocardial caspase 3/7 activity in LPS-challenged mice was significantly elevated at 6 hours after LPS treatment, which was markedly suppressed by ATE pretreatment (Figure 8C). However, there was no significant difference in serum TNF-α levels between the LPS group (8.2 ± 0.9 ng/mL) and the ATE plus LPS group (6.4 ± 0.4 ng/mL).

## Discussion

Our previous study demonstrated that systemic administration of 10 mg/kg ATE, a β_1_-AR antagonist, inhibited myocardial caspase 3/7 activation in LPS-challenged mice [19]. However, indirect effects of peripheral β_1_-AR blockade on systemic inflammation in endotoxemia cannot be ruled out, as it was reported that peripheral β_1_-AR blockade attenuated systemic inflammation and improved cardiac dysfunction induced by sepsis [25]. In order to overcome this problem and recognize the role of cardiomyocyte β_1_-AR activation in cardiomyocyte apoptosis during endotoxemia, we first isolated adult mouse ventricular myocytes and investigated the effects of DOB, a β_1_-AR agonist, on LPS-induced cardiomyocyte apoptosis. The results showed that LPS increased cardiomyocyte caspase-3/7 activity and apoptosis in a dose-dependent manner. Although 0.02 μM DOB did not significantly affect cardiomyocyte apoptosis, it markedly promoted 10 ng/mL LPS-induced cardiomyocyte apoptosis. Particularly, low nanograms per milliliter of LPS concentrations in plasma occur acutely in patients with septic shock and the direct effects of low doses of LPS on cardiomyocytes are clinically relevant [26,27]. Thus, we used 0.02 μM DOB and 10 ng/mL LPS to treat cardiomyocytes and further explore the mechanisms underlying the enhancing effects of DOB on LPS-induced cardiomyocyte apoptosis.

Several studies have demonstrated that LPS directly induced cardiomyocyte apoptosis via the death-receptor-dependent pathway that activates caspase-8 and mitochondria-dependent apoptotic pathway resulting in caspase-9 activation [28,29]. Therefore, we examined the effects of DOB or/and LPS treatment on the activities of caspase-8 and caspase-9 in adult cardiomyocytes, and found that 10 ng/mL LPS significantly increased caspase-8 activity. However, 10 ng/mL LPS failed to activate caspase-9; this result contrasts sharply with the findings from the previous studies demonstrating that 1 μg/mL LPS caused caspase-9 activation in cardiomyocytes [28,29]. This conflicting result might be associated with the difference in concentrations of LPS used in these studies. Moreover, exposure of cardiomyocytes to 0.02 μM DOB not only promoted caspase-8 activation, but also enhanced caspase-9 activity in LPS-treated cardiomyocytes. These findings suggest that LPS at clinically relevant concentrations induces cardiomyocyte apoptosis mainly via the death-receptor apoptotic pathway, and β_1_-AR activation promotes LPS-induced cardiomyocyte apoptosis through both the death-receptor and mitochondria-dependent pathways.

Accumulating evidence indicates that TNF-α-mediated caspase-8 activation plays a significant role in LPS-induced cardiomyocyte apoptosis [7,12,30]. The present study demonstrated that LPS increased TNF-α production in adult ventricular myocytes, which was markedly enhanced by DOB treatment. It is well-documented that NF-κB and MAPK activation mediate LPS-induced TNF-α expression in cardiomyocytes [31,32]. Thus, we examined NF-κB activity as well as IκBα, JNK and p38 MAPK phosphorylation, and found that LPS induced NF-κB activation as well as IκBα, JNK and p38 MAPK phosphorylation in cardiomyocytes, all of which were enhanced by DOB treatment.

These data suggest the possibility that β_1_-AR stimulation promotes LPS-induced TNF-α expression and caspase-8 activation maybe via enhancing IκBα, JNK and p38 MAPK phosphorylation in cardiomyocytes. As β_1_-AR couples to the stimulatory G protein, resulting in the activation of PKA [33], and PKA can activate JNK and p38 MAPK in the cardiomyocytes [32,33], we further examined the effects of KT5720, a cell-permeable PKA inhibitor, on cardiomyocytes challenged with LPS and DOB. The results showed that pretreatment with KT5720 abrogated the enhancement effects of DOB on p38 MAPK and JNK phosphorylation, but not on IκBα phosphorylation and caspase-8 activation in LPS-challenged cardiomyocytes. Evidently, β_1_-AR stimulation enhances LPS-induced TNF-α expression and caspase-8 activation mainly through promoting IκBα phosphorylation, rather than JNK and p38 MAPK phosphorylation in cardiomyocytes under our experimental conditions; this process is independent of PKA activation.

As mentioned above, β_1_-AR stimulation also strengthens LPS-induced cardiomyocyte apoptosis through activating the mitochondria-dependent apoptotic pathway. We observed that 10 ng/ml LPS treatment alone did not significantly affect cytosolic Cyt c, Bcl-2, mitochondrial Bax protein levels and mitochondrial membrane potential in cardiomyocytes. In contrast, co-stimulation with DOB resulted in a marked decrease in Bcl-2 protein level and mitochondrial membrane potential simultaneously with an increase in cytosolic Cyt c and mitochondrial Bax levels in LPS-treated cardiomyocytes. Previous studies have demonstrated that JNK-dependent activation of the mitochondrial death pathway is involved in β_1_-AR-stimulated cardiomyocyte apoptosis [33,34] and p38 MAPK has been found to mediate oxidative stress-triggered cardiomyocyte apoptosis [35]. Particularly, activation of p38 MAPK by oxidative stress results in the phosphorylation and degradation of Bcl-2 as well as the inactivation of its anti-apoptotic activity in cardiomyocytes [36], and p38 MAPK activation can also cause Bax translocation to mitochondria in cardiomyocytes [37]. Thus, it seems reasonable to speculate that β_1_-AR stimulation strengthens LPS-induced cardiomyocyte apoptosis maybe via increasing JNK and p38 MAPK phosphorylation, subsequently reducing Bcl-2 level, and in turn inducing Bax translocation to mitochondria, Cyt c release and caspase-9 activation. To test this hypothesis, the present study further investigated whether inhibition of PKA abolished the effects of β_1_-AR stimulation on caspase-9 activation, JNK and p38 MAPK phosphorylation as well as Bcl-2 protein level in LPS-treated cardiomyocytes. The data showed that pretreatment with PKA inhibitor not only blocked DOB-induced increase in caspase-9 activity as well as JNK and p38 MAPK phosphorylation, but also reversed DOB-provoked decrease in Bcl-2 protein level in LPS-treated cardiomyocytes. Taken together, these results suggest that PKA activation by β_1_-AR stimulation enhances JNK and p38MAPK phosphorylation, reduces Bcl-2 levels, increases Bax translocation to mitochondria, and in turn activates caspase-9 and promotes LPS-stimulated cardiomyocyte apoptosis.

On the other hand, it was reported that sustained (24-hour) β_1_-AR stimulation activates the PKA-independent Ca^2+^/ CaMKII signaling pathway and subsequently induces cardiomyocyte apoptosis [24,33,38]. β_1_-AR stimulation increases cytosolic Ca^2+^ concentration and activates CaMKII in cardiomyocytes, blockade of L-type calcium channel with NIF or inhibition of CaMKII activity fully attenuates cardiomyocyte apoptosis induced by β_1_-AR stimulation, and overexpression of active CaMKII leads to cardiomyocyte apoptosis through increased cytochrome c release [24,33,39]. Accordingly, we observed the effects of NIF on cytosolic Ca^2+^ concentration, CaMKII, caspase-3/7 and caspase-9 activation in DOB- and LPS-treated cardiomyocytes, and found that LPS not only increased cytosolic Ca^2+^ concentration, but also elevated CaMKII phosphorylation in cardiomyocytes, both of which were enhanced by DOB treatment. Furthermore, NIF pretreatment completely eliminated the effects of DOB on cytosolic Ca^2+^ concentration and CaMKII phosphorylation, and partly reversed the influence of DOB on caspase-9 and caspase-3/7 activities in LPS-treated cardiomyocytes. These data revealed that β_1_-AR activation enhanced LPS-induced cardiomyocyte apoptosis, at least in part, via increasing cytosolic calcium concentration and activating CaMKII. However, Zhang, *et al*. recently demonstrated that PKA inhibition alleviated cardiomyocyte apoptosis induced by β_1_-AR stimulation, by preventing cytosolic and sarcoplasmic reticulum Ca^2+^ overload and CaMKII activation [40]. Thus, it is worthy to note here that PKA-dependent activation of CaMKII may mediate the promoting effect of β_1_-AR stimulation on LPS-induced cardiomyocyte apoptosis. Future studies need to clarify this issue.

In order to confirm the current *in vitro* observations, we further examined the effect of ATE, a β_1_-AR antagonist, on myocardial apoptosis-associated molecules in LPS-challenged mice. Consistently, LPS challenge markedly upregulated myocardial TNF-α protein expression, increased myocardial JNK, p38 MAPK and CaMKII phosphorylation, reduced Bcl-2 protein level, and led to an increase in cytochrome c release and cardiomyocyte apoptosis in mice, all of which were reversed by pretreatment with ATE at a single dose of 10 mg/kg. In addition, ATE did not significantly affect serum TNF-α level in LPS-challenged mice. These findings confirm that β_1_-AR activation augments cardiomyocyte apoptosis during endotoxemia by upregulating TNF-α expression, enhancing JNK, p38 MAPK and CaMKII activation, reducing Bcl-2 protein level and increasing cytochrome c release in the myocardium.

## Conclusions

**Figure 9:**
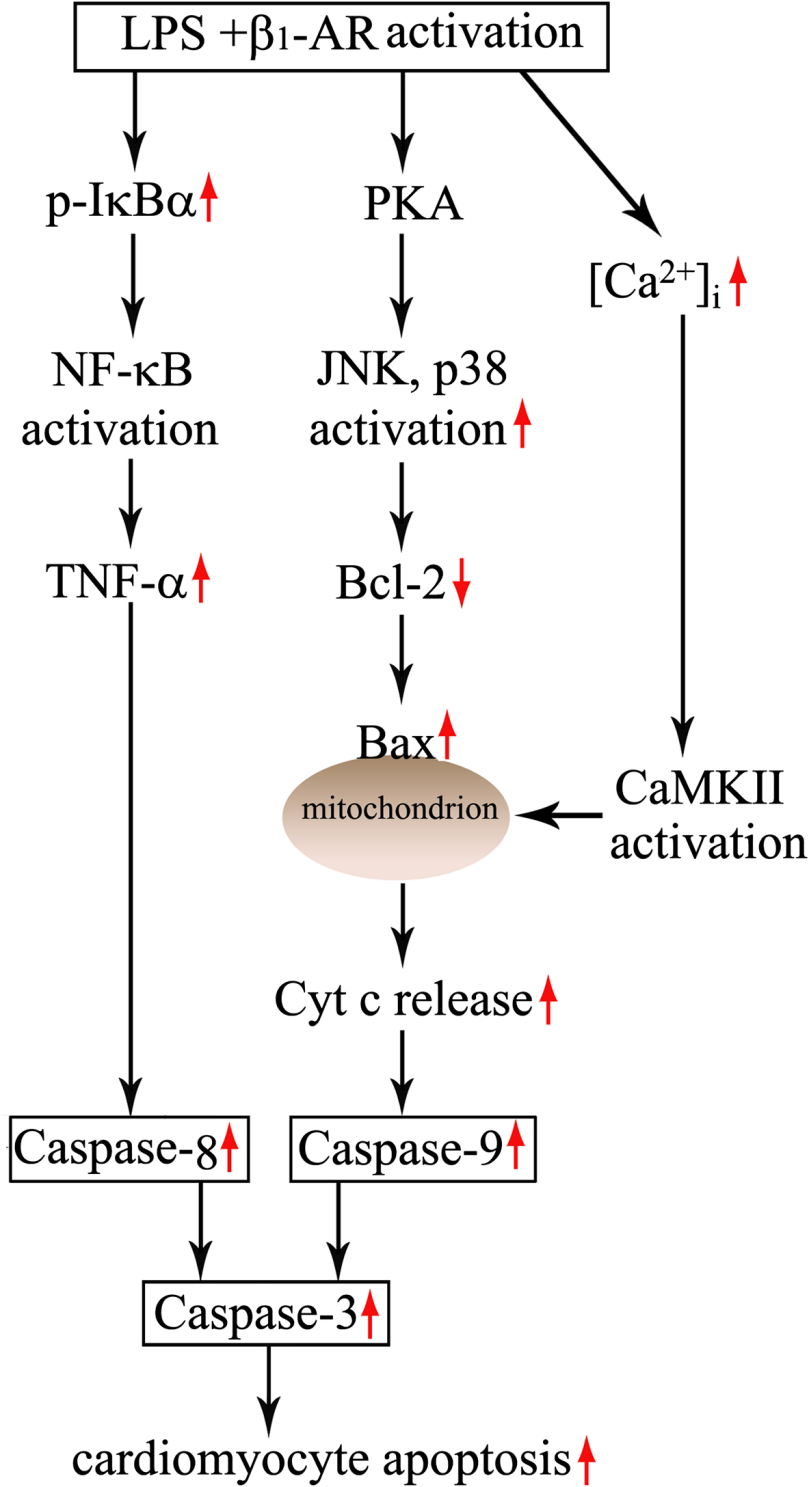
**Proposed signaling mechanisms for lipopolysaccharide (LPS)-induced cardiomyocyte apoptosis enhanced by β**_**1**_ **-AR activation.** Cardiomyocyte β_1_-AR activation augments IκBα phosphorylation and TNF-α expression, activates protein kinase A (PKA), enhances calmodulin-dependent protein kinase II (CaMKII) phosphorylation, and subsequently promotes LPS-induced cardiomyocyte apoptosis. Cardiomyocyte PKA activation enhances p38 mitogen-activated protein kinase (MAPK) and c-jun NH2-terminal kinase (JNK) phosphorylation, reduces Bcl-2 protein levels, and in turn leads to an increase in cytochrome c release and caspase-9 activity during LPS challenge.

To the best of our knowledge, the present study presents for the first time the direct evidence that cardiomyocyte β_1_-AR activation plays a pivotal role in cardiomyocyte apoptosis during endotoxemia, and cardiomyocyte β_1_-AR activation promotes LPS-induced cardiomyocyte apoptosis through activating PKA, increasing cytosolic Ca^2+^ concentration and CaMKII activation as well as enhancing IκBα phosphorylation and TNF-α expression in cardiomyocytes (Figure 9). These findings provide new insight into the mechanisms of sepsis-induced cardiomyocyte apoptosis and highlight the importance of β_1_-AR blocking in the treatment of myocardial dysfunction during sepsis.

## Key messages

- Cardiomyocyte β_1_-AR activation strengthens LPS-induced cardiomyocyte apoptosis through activating PKA and increasing cytosolic Ca^2+^ concentration and CaMKII activation as well as TNF-α expression in cardiomyocytes
- Cardiomyocyte β_1_-AR stimulation enhances LPS-induced cardiomyocyte IκBα phosphorylation and caspase-8 activation in a PKA-independent manner
- PKA activation by β_1_-AR stimulation augments JNK and p38 MAPK phosphorylation, reduces Bcl-2 level, and subsequently enhances Bax translocation to mitochondria, cytochrome c release and caspase-9 activation in LPS-challenged cardiomyocytes
- Cardiomyocyte β_1_-AR activation plays an important role in cardiomyocyte apoptosis during endotoxemia

## Abbreviations

AI: apoptotic index
AR: adrenoceptor
ATE: atenolol
CaMKII: calmodulin-dependent protein kinase II
Cyt: cytochrome
DAPI: 4',6-diamidino-2-phenylindole
DMEM: Dulbecco’s modified Eagle’s medium
DOB: dobutamine
ERK: extracellular signal-regulated kinase
GAPDH: glyceraldehyde-3-phosphate dehydrogenase
JNK: c-jun NH2-terminal kinase
LPS: lipopolysaccharide
MAPK: mitogen-activated protein kinase
NIF: nifedipine
PBS: phosphate-buffered saline
PKA: protein kinase A
TNF-α: tumor necrosis factor-α
TUNEL: terminal deoxynucleotidyl transferase-mediated dUTP nick-end-labeling
VDAC: voltage-dependent anion channel
